# Factors associated with the use of potentially inappropriate medication by elderly patients prescribed at hospital discharge

**DOI:** 10.31744/einstein_journal/2020AO4877

**Published:** 2019-10-22

**Authors:** Mariana Santos Magalhães, Fabiana Silvestre dos Santos, Adriano Max Moreira Reis

**Affiliations:** 1 Universidade Federal de Minas Gerais, Belo Horizonte, MG, Brazil.

**Keywords:** Potentially inappropriate medication list, Inappropriate prescribing, Age, Patient discharge, Drug therapy

## Abstract

**Objective:**

To analyze the frequency of use of potentially inappropriate medication prescribed to elderly at hospital discharge from a public hospital, considering the Brazilian Consensus on Potentially Inappropriate Medication for Elderly, and to identify the associated factors.

**Methods:**

Patients aged ≥60 years, admitted in clinical and geriatric units of a public hospital were invited to participate in the study. The information about the use of medicines was collected from the patient’s electronic record and through telephone contact. The Brazilian Consensus on Potentially Inappropriate Medication for Elderly was used to classify the medication, regardless of the clinical condition.

**Results:**

A total of 255 elders were included in this study. The frequency of use of potentially inappropriate medication by elderly was 58.4%. The potentially inappropriate medication use in elderly was positively associated with the presence of depression (odds ratio of 2.208) and polypharmacy (odds ratio of 2.495). The hospitalization in a geriatric unit showed an inverse association with the potentially inappropriate medication use in elderly (odds ratio of 0.513).

**Conclusion:**

The frequency of potentially inappropriate medication prescription to elderly upon hospital discharge was high. The presence of depression and polypharmacy were directly associated with use of potentially inappropriate medication in the elderly. Admission to the geriatric clinic has become a protection factor for the use of potentially inappropriate medication in elderly. Strategies to improve the elderly pharmacotherapy should implemented aiming at healthcare quality and safety in the transition of care.

## INTRODUCTION

Brazil is facing a steep increase of elderly individuals due to an accelerated demographic transition. From 1960 to 2008, the number of Brazilian individuals ≥60 years old increased seven fold, going from 3 million to 20 million.^1^ This increase in longevity brought about an elevated prevalence of chronic non-communicable diseases and increased use of medication.^2 , 3^

Data from the Brazilian National Survey on Access, Use and Promotion of Rational Use of Medicines (PNAUM - *Pesquisa Nacional sobre Acesso, Utilização e Promoção do Uso Racional de Medicamentos no* Brazil) show that approximately 93% of Brazilian elderly individuals use at least one continued use medication and 18% are on concomitant use of five or more drugs (polypharmacy),^3^ which puts them at risk of drug interactions,^4^ adverse effects,^4^ and being prescribed potentially inappropriate medications.^5^

Potentially inappropriate medications (PIMs) for elderly patients are defined as those that offer more risks than clinical benefits when safer and more effective alternatives are available.^6 , 7^ They should be avoided because of the risk of negative outcomes, such as falls and increases in health costs.^8^

Explicit criteria are paramount to evaluate the adequacy of medications prescribed to the elderly population.^9^ Such criteria consist of a list of medications that should be avoided in elderly patients based on literature reviews or a consensus among specialists.^9^ The most widely known and used criteria in clinical practice are The Beers criteria, from the American Geriatrics Society (AGS), and the Screening Tool of Older Persons’ Prescriptions (STOPP) from Ireland.^7^

The Brazilian Consensus of Potentially Inappropriate Medications for the Elderly (CBMPII - *Consenso Brasileiro de Medicamentos Potencialmente Inapropriados para Idosos* ) was published in 2017, and was the first explicit criteria validated to identify PIMs in Brazil.^7^ Having an explicit criteria that includes the medications available in the country gives us the ability to more accurately measure the use of PIMs, and elaborate educational strategies regarding the adequate and safe prescription of medications for the elderly.

Hospital admissions have a significant effect on PIMs used outside the hospital. Some studies comparing the prevalence of PIMs upon admission and discharge show that the time spent at hospital reduces the use of medications considered unsafe or unnecessary.^10 , 11^ However, PIM prevalence at discharge is still high and directly related to the number of prescribed medications.^5 , 10 , 12 , 13^

In Brazil, there are still few studies related to PIM use at hospital discharge considering the relevance and impact this issue presents to healthcare policies aimed at elderly individuals in the country.

## OBJECTIVE

To analyze the frequency of potentially inappropriate medications prescribed to elderly patients at hospital discharge in a public hospital, and identify associated factors.

## METHODS

### Study design and setting

This is a cross-sectional study conducted at a renowned public teaching hospital for civil servants in the Brazilian State of Minas Gerais. The hospital offers medium and high complexity care in the following specialties: general surgery, endocrinology, orthopedics and traumatology, ophthalmology, vascular surgery, gynecology and obstetrics, pediatrics, medical and geriatric clinic, among others.

### Study population and eligibility criteria

The selected sample was non-probabilistic and included elderly patients admitted to the hospital between April and November 2017. Elderly individuals were determined to be those ≥60 years old, as per the definition established by the World Health Organization (WHO) for developing countries, such as Brazil.

Eligibility criteria included elderly individuals admitted to the clinical and geriatric units between April 4^th^ and November 1^st^, 2017. Exclusion criteria included patients who died during hospital stay, were admitted for more than 60 days, left the hospital against medical advice or were lost to follow-up after discharge.

### Ethical considerations

This study was approved by the Research Ethics Committee from the *Universidade Federal de Minas Gerais* under protocol number 1.952.130, CAAE: 63612216.7.0000.5149. An informed consent form was signed by all the patients and/or their accompanying persons.

### Data collection

Patients admitted for more than 24 hours were identified through a report generated by the hospital admission system and later invited to participate in the study. An interview with the patients was conducted to retrieve demographic, functionality and clinical data, which were later complemented by information from the patient electronic record.

Information about the use of medications was collected from the electronic records and verified over the phone. To classify PIMs, we used the CBMPII criteria, regardless of the clinical condition.^7^

### Study variables

The dependent variable of the study was the frequency of PIMs prescribed upon discharge. Independent variables were divided into sociodemographic (sex, age, and marital status – reclassified as “has or does not have a partner”); clinical characteristics (unit of referral, diagnosis upon admission, and comorbidities – for which we used the Charlson comorbidity index – CCI);^14^ functional characteristics (vulnerability assessed by the Vulnerable Elders Survey – VES-13);^15^ pharmacotherapeutic characteristics (number of medications upon admission and discharge, pharmacotherapy complexity – measured by the Medication Regimen Complexity Index (MRCI) Brazilian version,^16^ stratified in high complexity: MRCI >16.5 yes or no, according to MRCI validation for the elderly in Brazil);^17^ and presence of polypharmacy (use of five or more medications).^18^

### Statistical analysis

Statistical analysis was done by determining the relative and absolute frequencies of categorical variables, and by determining the mean and standard deviation (SD), and/or the median and interquartile range (IQR) for numerical variables. Analysis of normality was done through the Shapiro-Wilk test. Numerical variables were dichotomized by the median. The association between the use of PIMs and independent variables was calculated using the χ^2^ test and Fisher’s exact test. Statistical significance level was established at p<0.05. Independent variables which presented an association with PIM use and obtained a p<0.20 in the univariate analysis were listed for the multivariate logistic regression. To obtain the final model, we used the backward stepwise method and variables kept a p<0.05. For the final model’s adequacy assessment, we used the Hosmer-Lemeshow test (adjusted when p>0.05).

## RESULTS

Clinical and sociodemographic characteristics of the 255 elderly individuals in the study are described in table 1 . The sample had a majority of women (57.3%), median age of 75 years (IQR=13) and most participants did not have a partner (50.9%).

**Table 1:** Clinical and sociodemographic characteristics

Sociodemographic characteristics	
Age, years	75 [13]
Female, sex	146 (57.3)
Does not have a partner	130 (50.9)
Clinical characteristics	
VES-13^*^ score	5 (6.0)
Charlson comorbidity index	5 (2.0)
Diagnosis upon admission	
Respiratory disease	64 (25.1)
Diseases of the genitourinary system	43 (16.9)
Circulatory diseases	30 (11.8)
Symptoms, signs and abnormal findings from laboratory and clinical tests, not classified elsewhere	25 (9.8)
Some infectious and parasitic diseases	21 (8.2)
Digestive disorders	18 (7.1)
Mental and behavioral disorders	14 (5.5)
Endocrine, nutritional and metabolic diseases	11 (4.3)
Others	40 (15.6)
Pharmacotherapy	
Polypharmacy (≥5 medications)	174 (68.2)
Number of medications used upon discharge	6 [4]
Number of medications used upon admission	5 [5]
Patients using PIMs	149 (58.4)
Maximum number of PIMs per patient	4 (1-4)

Results expressed as median [interquartile range]; n (%) or n (minimum – maximum). * Individuals with a score ≥3 have 4.2 times more risk of functional decline and death in two years compared to those with lower scores. VES-13: Vulnerable Elders Survey; PIMs: potentially inappropriate medications for the elderly.

The median vulnerability score was 5 points (IQR=6). The disease burden assessed by the CCI presented a median of 5 (IQR=2). The main clinical diagnoses upon admission were respiratory diseases (25.1%), diseases of the genitourinary system (16.9%) and circulatory diseases (11.8%).

Pharmacotherapy characteristics showed a median of medications used upon admission of 5 (IQR=5) and upon discharge of 6 (IQR=5). Polypharmacy was present in most cases (68.2%).

Of the participants, 58.4% were using one or more PIMs upon discharge. The minimum and maximum numbers of PIMs prescribed ranged between 1 and 4, respectively.

Proton pump inhibitors (PPI) were the most frequently used PIM (43.8%). Other frequently used PIMs were benzodiazepines (14.9%); second generation antipsychotics (14.9%); phenobarbital (3.8%) and haloperidol (3.4%). The distribution of the frequency of PIM use is shown in table 2 .

**Table 2:** Potentially inappropriate medications for the elderly (PIMs), regardless of clinical condition, and according to the Brazilian Consensus of Potentially Inappropriate Medications for the Elderly

Criteria/PIM	n (%)
Proton pump inhibitors (>8 weeks)	91 (43.8)
Omeprazole	77 (37.0)
Pantoprazole	14 (6.7)
Benzodiazepines	31 (14.9)
Clonazepam	21 (10.1)
Lorazepam	8 (3.8)
Bromazepam	8 (3.8)
Alprazolam	1 (0.5)
Second generation antipsychotics	31 (14.9)
Quetiapine	17 (8.2)
Risperidone	12 (5.8)
Clozapine	2 (1.0)
Barbiturates	
Phenobarbital	8 (3.8)
First generation antipsychotics	
Haloperidol	7 (3.4)
Antiarrhythmic classes Ia, Ic, III	
Amiodarone	6 (2.9)
Non-benzodiazepine hypnotics for >90 days	
Zolpidem	4 (1.9)
Tertiary tricyclic antidepressants	4 (1.9)
Nortriptyline	3 (1.4)
Amitriptyline	1 (0.5)
Nitrofurantoin	4 (1.9)
Prolonged use of strong opioids as first line therapy for mild/moderate pain	4 (1.9)
Morphine	3 (1.4)
Methadone	1 (0.5)
Glibenclamide	3 (1.4)
Central alpha agonists	
Clonidine	3 (1.4)
Metoclopramide	3 (1.4)
Prolonged use of colchicine for gout	2 (1.0)
Nonsteroidal anti-inflammatory drugs (non-selective COX)	2 (1.0)
Ibuprofen	1 (0.5)
Naproxen	1 (0.5)
Anti-histamínicos de primeira geração	
Dexchlorpheniramine	2 (1.0)
Antiparkinsonian agents with strong anticholinergic action	
Biperiden	1.0 (0.5)
Mineral oil (oral)	1.0 (0.5)
Muscle Relaxant	
Cyclobenzaprine	1.0 (0.5)

COX: cyclooxygenase.

The use of PIMs was associated to sex (female); unit of referral (geriatrics); presence of diseases (hypertension and depression); polypharmacy and the high level of pharmacotherapy complexity, as shown by the univariate statistical analysis ( Table 3 ).

**Table 3:** Univariate and multivariate analysis of factors associated to the use of potentially inappropriate medications among elderly patients admitted to a public hospital in Minas Gerais

Variable	PIM use					
	Frequency*		Univariate analysis		Multivariate analysis*	
		
	Yes	No	Odds ratio	p value	Odds ratio	p value
	n (%)	n (%)	(95%CI)		(95%CI)	
Sociodemographic						
Sex						
Female	94 (63.1)	52 (49.1)	1.775 (1.070-2.943)	0.026	-	-
Male	55 (36.9)	54 (50.9)	1			
Age						
≥75	84 (56.4)	56 (52.8)	1.154 (0.700-1.903)	0.575	-	-
<75	65 (43.6)	50 (47.2)	1			
Clinical						
Unit of referral						
Geriatrics	66 (44.3)	31 (29.2)	0.520 (0.306-0.882)	0.015	0.513 (0.295-0.892)	0.018
Other clinics	83 (55.7)	75 (70.8)	1			
Stroke						
Yes	22 (14.8)	15 (14.2)	1.051 (0.517-2.136)	0.891	-	-
No	127 (85.2)	91 (85.8)	1			
Heart failure						
Yes	24 (16.1)	14 (13.2)	1.262 (0.619-2.571)	0.522	-	-
No	125 (83.9)	92 (86.8)	1			
COPD						
Yes	20 (13.4)	14 (13.2)	1.019 (0.489-2.122)	0.960	-	-
No	129 (86.6)	92 (86.8)	1			
Cancer						
Yes	19 (12.8)	10 (9.4)	1.403 (0.624-3.154)	0.411	-	-
No	130 (87.2)	96 (90.6)	1			
*Diabetes mellitus*						
Yes	63 (42.3)	47 (44.3)	0.920 (0.556-1.520)	0.744	-	-
No	86 (57.7)	59 (55.7)	1			
Pneumonia						
Yes	32 (21.5)	21 (19.8)	1.107 (0.597-2.052)	0.747	-	-
No	117 (78.5)	85 (80.2)	1			
Dementia						
Yes	51 (34.2)	28 (26.4)	1.450 (0.838-2.509)	0.184	-	-
No	98 (65.8)	78 (73.6)	1			
Chronic kidney disease						
Yes	31 (20.8)	14 (13.2)	1.726 (0.868-3.433)	0.117	-	-
No	118 (79.2)	92 (86.8)	1			
Atrial fibrillation						
Yes	12 (8.1)	9 (8.5)	0.944 (0.383-2.328)	0.900	-	-
No	137 (91.9)	97 (91.5)	1			
Hypertension						
Yes	113 (75.8)	68 (64.2)	1.754 (1.016-3.029)	0.043	-	-
No	36 (24.2)	38 (35.8)	1			
Acute myocardial infarction						
Yes	14 (9.4)	5 (4.7)	2.095 (0.731-6.005)	0.16^†^	-	-
No	135 (90.6)	101 (95.3)	1			
Parkinsonism						
Yes	7 (4.7)	0 (0.0)	1.746 (1.568-1.945)	0.44^†^	-	-
No	142 (95.3)	106 (100)				
Depression						
Yes	35 (23.5)	11 (10.4)	2.652 (1.278-5.503)	0.007	2.208 (1.035-4.707)	0.040
No	114 (76.5)	95 (89.6)	1			
Charlson comorbidity index						
≥5	85 (57.0)	58 (54.7)	1.099 (0.666-1.815)	0.712	-	-
<5	64 (43.0)	48 (45.3)				
Functionality						
VES-13						
≥5	85 (57.0)	51 (48.1)	1.432 (0.868-2.362)	0.159	-	-
<5	64 (43.0)	55 (51.9)	1			
Pharmacotherapy						
Polypharmacy 5						
Yes	114 (76.5)	60 (56.6)	2.497 (1.456-4.283)	0.001	2.495 (1.431-4.349)	0.001
No	35 (23.5)	46 (43.4)	1			
High level of pharmacotherapy complexity						
Yes	93 (62.4)	40 (37.7)	2.740 (1.639-4.581)	0.000	-	-
No	56 (37.6)	66 (62.3)	1			

^†^ p value was calculated by Fisher’s exact test; * Hosmer-Lemeshow goodness of fit test: degrees of freedom = 5; χ^2^ = 2.40; p=0.79. PIM: Potentially Inappropriate Medications for the Elderly; COPD: chronic obstructive pulmonary disease; VES-13: Vulnerable Elders Survey.

In the final logistic regression model of the multivariate analysis, the unit of referral (geriatrics), presence of depression and polypharmacy presented a positive association with PIM use. Admission to the geriatric clinic showed an inverse association with PIM use.

## DISCUSSION

The prevalence of PIM at hospital discharge among the participating elderly individuals was high and was associated to depression and the use of polypharmacy. Admission to the geriatric clinic showed an inverse association with the use of PIM. The study expanded our knowledge about the use of PIMs upon hospital discharge, because a previous investigation conducted in Brazil was limited to patients with a history of cardiovascular disease. Our study also helped to identify associated factors. The frequency of PIM use in our study was higher to the one recorded at a teaching hospital in São Paulo, Brazil^19^ (13.9%) and to some in European countries, which varied between 23.5 and 48.0%.^12 , 20 , 21^ A Brazilian study has also demonstrated a high prevalence of PIMs during hospital stay,^22^ showing the importance of preventing and reducing the use of these medications from the moment the patient is admitted. Results must be compared with caution because of the methodological differences and the explicit criterion used to identify the PIMs.

This study is the first to determine the frequency of PIM use at discharge through the CBMPII. Studies that use explicit criteria that were established to reflect the reality of a certain country are very important – they offer a better description of the profile of PIM use and better identification of associated factors. Moreover, they provide the basis for safe clinical practice and for the adoption of public policies to reduce complications related to pharmacotherapy in the elderly population of the country. The CBMPII presented good conformity with the international criteria AGS Beers 2015 and the European Union List of Potentially Inappropriate Medications (EU)(7)-PIM List), which shows its adequacy for pharmacoepidemiologic studies and clinical practice.^23^

One relevant finding was the high prevalence of PPI prescribed at discharge for elderly patients. This is in accordance with a research conducted at a community hospital in Japan, using the AGS Beers 2015, whose PPI prescription frequency accounted for about 38% of PIMs used at discharge.^11^ The prolonged use (>8 weeks) of PPIs increases the risk of *Clostridium difficile* infection,^6^ and of fractures, reduces bone mineral density,^6 , 7^ increases the risk of dementia and renal failure.^7^ It is noteworthy that a Brazilian study with community elders also showed a high prevalence of PPI use.^23^ Such findings reinforce the importance of developing manuals about the adequate and safe prescription of PPI and guidelines for the deprescription of this therapeutic class.

We also highlight the elevated frequency of prescriptions with benzodiazepines, whose use among the elderly population is well established. In Japan, about 30% of patients were prescribed benzodiazepines at discharge.^11^ In the United States, a study that evaluated outpatient prescriptions for 10 years showed that benzodiazepines were prescribed to elderly patients in 12.45% of appointments.^24^ The use of benzodiazepines, the long-acting variety in particular,^6^ by elderly individuals is associated to negative outcomes such as falls, fractures, car accidents, cognitive impairment, and *delirium* .^6 , 7^

In this study, among the inappropriate medications that act on the nervous system, we found a high prevalence of antipsychotics – typical (haloperidol) and atypical (quetiapine, risperidone, and clozapine) – which is similar to findings from studies done outside Brazil.^11 - 22^ This therapeutic class is often prescribed for behavioral and psychological symptoms of dementia in elders. Antipsychotics are considered PIMs because they increase the risk of stroke,^6 , 7^ cognitive decline^6^ and mortality when used for a prolonged period by elderly patients.^6^

In elderly patients, the use of barbiturates must be avoided because of their addictive potential and tolerance development for sleep induction, and the risk of poisoning due to their narrow therapeutic index.^6 , 7^ The increase in permeability of the blood-brain barrier, associated to other physiological modifications of the aging process, elevates barbiturate-sensitivity,^25^ which justifies strong caution when prescribing barbiturates. Their use among the elderly individuals included in the study was restricted to phenobarbital and its frequency was reduced. Therefore, strategies to avoid PIM prescription and use should be implemented. The assistance provided by clinical pharmacists in association with a multidisciplinary team of geriatric care has been described as an effective clinical practice to improve prescription appropriateness and ensure the pharmacotherapy safety of elders at admission and discharge.^26^

Polypharmacy showed a positive association to the use of PIMs at admission, which is similar to results from other studies.^5 - 13^ A Spanish study showed that for each additional medication at discharge, there is a 14 to 15% increase in the risk of PIM use.^5^ Despite its negative effects, polypharmacy if often necessary. The treatment of multiple diseases requires the concomitant use of several medications and polypharmacy is a strategy for the adequate maintenance of elderly individuals’ pharmacotherapy.^27^

We also found a positive association between depression and the use of PIMs. This finding can be attributed to the high prevalence of psychotropic drugs among PIMs used by the elderly population. A French study that assessed hospitalized elderly patients found an association between previous use of psychotropic drugs (2 weeks before admission) and depression.^28^ In Brazil, depression was the variable most strongly associated to the use of psychotropic drugs by elders in the metropolitan area of the Brazilian city of Belo Horizonte.^29^

Admission to the geriatric clinic was shown to be a protective factor against the use of PIMs within the studied population. The care provided in the specialized geriatric unit is associated to clinical benefits, such as a reduction of polypharmacy,^30^ simplification of dose regimens^30^ and a decreased use of PIMs at discharge.^10^ This result reinforces the importance of a global and multidisciplinary approach to elderly patient care to recognize the specificities and demands of the aging process, especially those associated to the use of medications.^26^

This study presents similar results to those found in the international literature and represents a landmark in pharmacoepidemiologic studies with elderly individuals in Brazil because it uses a national criterion. However, it does present limitations that should be addressed. First, we do not identify the period of use or dosage of the medications, which could impact the measurement of certain criteria that define PIMs. Moreover, the evaluation of PIM use without considering the clinical condition may have overestimated the usage frequency of PIMs. The second limitation is that we cannot extrapolate our results because the study was conducted in only one hospital whose patients are all civil servants.

## CONCLUSION

The frequency of potentially inappropriate medications prescription at discharge at the studied hospital was high. Polypharmacy and depression were positively associated to the use of potentially inappropriate medications. Admission to geriatric units presented an inverse association to the use of potentially inappropriate medications by elderly patients. Strategies to improve pharmacotherapy for elderly patients should be implemented to improve the quality of assistance and safety during the transition of care.

## References

[1] 1. Veras RP. Population aging today: demands, challenges and innovations. Rev Saude Publica. 2009;43(3):548-54. Review. 10.1590/s0034-89102009000300020 19377752

[2] 2. Veras RP. Um modelo em que todos ganham: mudar e inovar, desafios para o enfrentamento das doenças crônicas entre os idosos. Acta Sci. 2012; 34(1):3-8.

[3] 3. Ramos LR, Tavares NU, Bertoldi AD, Farias MR, Oliveira MA, Luiza VL, et al. Polypharmacy and Polymorbidity in Older Adults in Brazil: a public health challenge. Rev Saude Publica. 2016;50(Suppl 2):9s. 10.1590/S1518-8787.2016050006145 PMC5157903 27982377

[4] 4. Maher RL, Hanlon J, Hajjar ER. Clinical consequences of polypharmacy in elderly. Expert Opin Drug Saf. 2014;13(1):57-65. Review. 10.1517/14740338.2013.827660 PMC3864987 24073682

[5] 5. Hudhra K, García-Caballos M, Casado-Fernandez E, Jucja B, Shabani D, Bueno-Cavanillas A. Polypharmacy and potentially inappropriate prescriptions identified by Beers and STOPP criteria in co-morbid older patients at hospital discharge. J Eval Clin Pract. 2016;22(2):189-93. 10.1111/jep.12452 26399173

[6] 6. By the American Geriatrics Society 2015 Beers Criteria Update Expert Panel. American Geriatrics Society 2015 Updated Beers Criteria for Potentially Inappropriate Medication Use in Older Adults. J Am Geriatr Soc. 2015;63(11): 2227-46. 10.1111/jgs.13702 26446832

[7] 7. Oliveira MG, Amorim WW, Borja-Oliveira CR, Coqueiro HL, Gusmão LC, Passos LC. Consenso brasileiro de medicamentos potencialmente inapropriados para idosos. Geriatr, Gerontol Aging. 2016;10(4):168-81.

[8] 8. Stockl KM, Le L, Zhang S, Harada AS. Clinical and economic outcomes associated with potentially inappropriate prescribing in the elderly. Am J Manag Care. 2010;16(1):e1-10. 20059286

[9] 9. Jansen PA, Brouwers JR. Clinical pharmacology in old persons. Scientifica (Cairo). 2012;2012:723678. Review. 10.6064/2012/723678 PMC3820465 24278735

[10] 10. Laroche ML, Charmes JP, Nouaille Y, Fourrier A, Merle L. Impact of hospitalisation in an acute medical geriatric unit on potentially inappropriate Medication Use. Drugs Aging. 2006;23(1):49-59. 10.2165/00002512-200623010-00005 16492069

[11] 11. Komagamine J. Prevalence of potentially inappropriate medications at admission and discharge among hospitalised elderly patients with acute medical illness at a single centre in Japan: a retrospective cross-sectional study. BMJ Open. 2018;8(7):e021152. 10.1136/bmjopen-2017-021152 PMC6059264 30030316

[12] 12. Galán Retamal C, Garrido Fernández R, Fernández Espínola S, Ruiz Serrato A, Gárcia Ordóñez M, Padilla Marín V. Prevalencia de medicación potencialmente inapropiada en pacientes ancianos hospitalizados utilizando criterios explícitos. Farm Hosp. 2014;38(4):305-16. Spanish. 10.7399/fh.2014.38.4.1148 25137164

[13] 13. Counter D, Millar JW, McLay JS. Hospital readmissions, mortality and potentially inappropriate prescribing: a retrospective study of older adults discharged from hospital. Br J Clin Pharmacol. 2018;84(8):1757-63. 10.1111/bcp.13607 PMC6046509 29744901

[14] 14. Charlson ME, Pompei P, Ales KL, MacKenzie CR. A new method of classifying prognostic comorbity in longitudinal studies: development and validation. J Chronic Dis. 1987;40(5):373-83. 10.1016/0021-9681(87)90171-8 3558716

[15] 15. Saliba D, Elliott M, Rubenstein LZ, Solomon DH, Young RT, Kamberg CJ, et al. The Vulnerable Elders Survey: a tool for identifying vulnerable older people in the community. J Am Geriatr Soc. 2001;49(12):1691-9. 10.1046/j.1532-5415.2001.49281.x 11844005

[16] 16. Melchiors AC, Correr CJ, Fernández-Llimos F. Tradução e validação para o português do Medication Regimen Complexity Index. Arq. Bras. Cardiol. 2007;89(4):210-18. 10.1590/s0066-782x2007001600001 17992376

[17] 17. Pantuzza LL, Ceccato MD, Silveira MR, Pinto IV, Reis AM. Validation and standardization of the Brazilian version of the Medication Regimen Complexity Index for older adults in primary care. Geriatr Gerontol Int. 2018;18(6):853-9. 10.1111/ggi.13261 29380500

[18] 18. Jyrkkä J, Enlund H, Korhonen MJ, Sulkava R, Hartikainen S. Patterns of drug use and factors associated with polypharmacy and excessive polypharmacy in elderly persons: results of the Kuopio 75+ study: a cross-sectional analysis. Drugs Aging. 2009;26(6):493-503. 10.2165/00002512-200926060-00006 19591524

[19] 19. Mori AL, Carvalho RC, Aguiar PM, de Lima MG, Rossi MD, Carrillo JF, et al. Potentially inappropriate prescribing and associated factors in elderly patients at hospital discharge in Brazil: a cross-sectional study. Int J Clin Pharm. 2017;39(2):386-93. 10.1007/s11096-017-0433-7 28188508

[20] 20. Hudhra K, Beçi E, Petrela E, Xhafaj D, García-Caballos M, Bueno-Cavanillas A. Prevalence and factors associated with potentially inappropriate prescriptions among older patients at hospital discharge. J Eval Clin Pract. 2016;22(5):707-13. 10.1111/jep.12521 27001470

[21] 21. Pasina L, Djade CD, Tettamanti M, Franchi C, Salerno F, Corrao S, et al. Prevalence of potentially inappropriate medications and risk of adverse clinical outcome in a cohort of hospitalized elderly patients: results from the REPOSI Study. J Clin Pharm Ther. 2014;39(5):511-5. 10.1111/jcpt.12178 24845066

[22] 22. Ulbrich AH, Cusinato CT, Guahyba RS. Medicamentos potencialmente inapropriados (MPIS) para idosos: prevalência em um hospital terciário do Brasil. Rev Bras Farm Hosp Serv Saúde. 2017;8(3):14-18.

[23] 23. Almeida TA, Reis EA, Pinto IV, Ceccato MD, Silveira MR, Lima MG, et al. Factors associated with the use of potentially inappropriate medications by older adults in primary health care: An analysis comparing AGS Beers, EU(7)-PIM List, and Brazilian Consensus PIM criteria. Res Social Adm Pharm. 2019;15(4):370-7. 10.1016/j.sapharm.2018.06.002 29934277

[24] 24. Marra EM, Mazer-Amirshahi M, Brooks G, van den Anker J, May L, Pines JM. Benzodiazepine Prescribing in Older Adults in U.S. Ambulatory Clinics and Emergency Departments (2001-10). J Am Geriatr Soc. 2015;63(10):2074-81. 10.1111/jgs.13666 26415836

[25] 25. Hughes SG. Prescribing for the elderly patient: why do we need to exercise caution? Br J Clin Pharmacol. 1998;46(6):531-3. 10.1046/j.1365-2125.1998.00842.x PMC1873806 9862240

[26] 26. Spinewine A, Swine C, Dhillon S, Lambert P, Nachega JB, Wilmotte L, et al. Effect of a Collaborative Approach on the Quality of Prescribing for Geriatric Inpatients: a randomized, Controlled Trial. J Am Geriatr Soc. 2007;55(5):658-65. 10.1111/j.1532-5415.2007.01132.x 17493184

[27] 27. Shah BM, Hajjar ER. Polypharmacy, adverse drug reactions, and geriatric Syndromes. Clin Geriatr Med. 2012;28(2):173-86. 10.1016/j.cger.2012.01.002 22500537

[28] 28. Prudent M, Dramé M, Jolly D, Trenque T, Parjoie R, Mahmoudi R, et al. Potentially Inappropriate Use of Psychotropic Medications in Hospitalized Elderly Patients in France: cross-sectional analysis of the prospective, multicentre SAFEs cohort. Drugs Aging. 2008;25(11):933-46. 10.2165/0002512-200825110-00004 18947261

[29] 29. Abi-Ackel MM, Lima-Costa MF, Castro-Costa E, Loyola FA. Psychotropic drug use among older adults: prevalence and associated factors. Rev Bras Epidemiol. 2017;20(1):57-69. 10.1590/1980-5497201700010005 28513794

[30] 30. Kruse W, Rampmaier J, Frauenrath-Volkers C, Volkert D, Wankmüller I, Micol W, et al. Drug-prescribing patterns in old age. A study of the impact of hospitalization on drug prescriptions and follow-up survey in patients 75 years and older. Eur J Clin Pharmacol. 1991;41(5):441-7. 10.1007/BF00626366 1761071

