# Supplementary material for: Factors associated with the use of potentially inappropriate medication by elderly patients prescribed at hospital discharge
Source: Einstein (Sao Paulo). 2019 Oct 22;18:eAO4877. doi: 10.31744/einstein_journal/2020AO4877 (PMC6896600; doi:10.31744/einstein_journal/2020AO4877)
Supplement: Translation

# Translation: Fatores associados ao uso de medicamentos potencialmente inapropriados para idosos na alta hospitalar

**Authors:** Mariana Santos Magalhães, Fabiana Silvestre dos Santos, Adriano Max Moreira Reis

**Affiliations:** 1 Universidade Federal de Minas Gerais, Belo Horizonte, MG, Brasil.

**Keywords:** Lista de medicamentos potencialmente inapropriados, Prescrição inapropriada, Idoso, Alta do paciente, Tratamento farmacológico

## Abstract

**Objetivo:**

Analisar a frequência de uso de medicamentos potencialmente inapropriados para idosos na prescrição de alta hospitalar de idosos de um hospital público, considerando o Consenso Brasileiro de Medicamentos Potencialmente Inapropriados para Idosos, e identificar os fatores associados.

**Métodos:**

Pacientes com idade ≥60 anos, internados nas unidades de clínica médica e geriátrica de um hospital público, foram convidados para participar do estudo. As informações sobre o uso de medicamentos foram coletadas do prontuário eletrônico do paciente e confirmadas por contato telefônico. O Consenso Brasileiro de Medicamentos Potencialmente Inapropriados para Idosos independente da condição clínica foi utilizado para a classificação dos medicamentos.

**Resultados:**

Foram incluídos no estudo 255 idosos. A frequência de uso de medicamentos potencialmente inapropriados para idosos foi de 58,4%. O uso de medicamentos potencialmente inapropriados para idosos foi associado positivamente à presença de depressão (razão de chance de 2,208) e polifarmácia (razão de chance de 2,495). A internação em unidade de geriatria apresentou associação inversa com uso de medicamentos potencialmente inapropriados para idosos (razão de chance de 0,513).

**Conclusão:**

A frequência de prescrição de medicamentos potencialmente inapropriados para idosos na alta hospitalar foi alta. Depressão e polifarmácia estiveram diretamente associadas ao uso de medicamentos potencialmente inapropriados para idosos. Ficar internado na clínica geriátrica mostrou-se fator protetor para uso de medicamentos potencialmente inapropriados para idosos. Estratégias para melhorar a farmacoterapia do idoso devem ser implementadas, visando à qualidade assistencial e à segurança na transição do cuidado.

## INTRODUÇÃO

O forte crescimento da população idosa brasileira resulta de uma transição demográfica acelerada. De 1960 a 2008, o número de brasileiros com idade ≥60 anos aumentou cerca de sete vezes, passando de 3 para 20 milhões.^1^ O aumento da longevidade é seguido por um incremento crescente da prevalência das doenças crônicas não transmissíveis e do uso de medicamentos.^2 , 3^

Dados da Pesquisa Nacional sobre Acesso, Utilização e Promoção do Uso Racional de Medicamentos no Brasil (PNAUM) revelam que aproximadamente 93% dos idosos brasileiros utilizam ao menos um medicamento cronicamente e 18% estão em uso de polifarmácia (uso concomitante de cinco ou mais medicamentos).^3^ O uso de múltiplos medicamentos pelos idosos predispõe a interações medicamentosas,^4^ eventos adversos a medicamentos^4^ e prescrição de medicamentos potencialmente inapropriados.^5^

Os medicamentos potencialmente inapropriados para idosos (MPII) são definidos como aqueles cujo risco de uso é maior que os benefícios clínicos proporcionados, quando alternativas mais seguras e efetivas estão disponíveis.^6 , 7^ Sua prescrição deve ser evitada, devido ao elevado potencial para provocar desfechos negativos, como quedas e aumento dos custos em saúde.^8^

Os critérios explícitos são instrumentos fundamentais na avaliação da adequação da prescrição de medicamentos para a população geriátrica.^9^ Consistem basicamente em uma lista de medicamentos que devem ser evitados em idosos, obtida a partir de revisões de literatura ou consenso de especialistas.^9^ O norte-americano Beers, da *American Geriatrics Society* (AGS), e o irlandês *Screening Tool of Older Persons’ Prescriptions* (STOPP) são os critérios explícitos mais conhecidos e utilizados na prática clínica.^7^

Em 2017, foi publicado o Consenso Brasileiro de Medicamentos Potencialmente Inapropriados para Idosos (CBMPII), primeiro critério explícito validado para a identificação de MPII no Brasil.^7^ A disponibilidade de um critério explícito abrangendo os medicamentos disponíveis no país permite mensuração mais exata da utilização de MPII e contribui para o desenvolvimento de ações educativas direcionadas à prescrição adequada e segura de medicamentos para idosos.

A hospitalização tem efeito importante no uso de MPII no regime ambulatorial. Alguns estudos que compararam a prevalência de MPII na admissão e na alta hospitalar demonstram que a hospitalização contribuiu para a redução de medicamentos considerados inseguros ou desnecessários.^10 , 11^ Contudo, a prevalência de MPII na alta hospitalar ainda é elevada e relacionada diretamente ao número de medicamentos prescritos.^5 , 10 , 12 , 13^

No Brasil, ainda são incipientes os estudos relacionados ao uso de MPII na alta hospitalar, considerando a relevância do tema e os impactos nas políticas de saúde direcionadas aos idosos do país.

## OBJETIVO

Analisar a frequência de uso de medicamentos potencialmente inapropriados para idosos na prescrição de alta hospitalar de idosos de um hospital público e identificar seus fatores associados.

## MÉTODOS

### Delineamento e local do estudo

Estudo transversal, realizado em um hospital público, de ensino, referência no atendimento de servidores públicos do Estado de Minas Gerais. O hospital realizava atendimentos de média e alta complexidade nas clínicas de cirurgia geral, endocrinologia, ortopedia e traumatologia, oftalmologia, cirurgia vascular, ginecologia e obstetrícia, pediatria, clínica médica e geriátrica, entre outras especialidades.

### População do estudo e critérios de elegibilidade

A amostra selecionada foi não probabilística e compreendeu idosos internados no hospital de abril a novembro de 2017. Para a definição de idoso, utilizou-se o critério estabelecido pela Organização Mundial da Saúde (OMS) para países em desenvolvimento, como o Brasil, que define idoso como indivíduo com idade ≥60 anos.

Os critérios de elegibilidade incluíram idosos internados nas unidades de clínica médica e geriátrica, durante o período de 4 de abril a 1° de novembro de 2017. Foram excluídos pacientes que foram a óbito durante a internação, tiveram internação superior a 60 dias, evadiram do hospital ou perdas de contato após a alta.

### Considerações éticas

O estudo foi aprovado pelo Comitê de Ética e Pesquisa da Universidade Federal de Minas Gerais sob parecer número 1.952.130, CAAE: 63612216.7.0000.5149. A participação na pesquisa foi precedida pela assinatura do Termo de Consentimento Livre e Esclarecido pelos pacientes e/ou acompanhantes.

### Coleta de dados

Os pacientes internados por período superior a 24 horas foram identificados por meio do relatório informatizado do sistema de internação do hospital investigado e, posteriormente, convidados a participar do estudo. Realizou-se entrevista com o idoso, para coleta de dados demográficos, de funcionalidade e clínicos. Os dados clínicos foram complementados com as informações presentes no prontuário eletrônico do paciente.

As informações sobre o uso de medicamentos foram coletadas do prontuário eletrônico e confirmadas por contato telefônico. Para a classificação dos MPII, utilizou-se como critério o CBMPII, independente da condição clínica.^7^

### Variáveis do estudo

A variável dependente do estudo foi a frequência de MPII prescritos na alta hospitalar.

As variáveis independentes foram divididas em sociodemográficas (sexo, idade e estado civil − reclassificado em tem ou não companheiro); características clínicas (internação de origem, diagnóstico na admissão e presença de comorbidades − para a qual foi determinado o índice de comorbidade de Charlson − ICC);^14^ características funcionais (vulnerabilidade avaliada pelo instrumento *Vulnerable Elders Survey* − VES-13);^15^ características farmacoterápicas (número de medicamentos na admissão e na alta hospitalar, complexidade da farmacoterapia − mensurada pelo *Medication Regimen Complexity Index* (MRCI), versão brasileira,^16^ estratificada em alta complexidade: MRCI >16,5 sim ou não, segundo a normatização do MRCI para idosos no Brasil)^17^ e presença de polifarmácia (uso de cinco ou mais medicamentos).^18^

### Análise estatística

A análise estatística foi realizada determinando-se a frequência relativa e absoluta das variáveis categóricas, a média e desvio padrão (DP), e/ou a mediana e amplitude interquatil (IQR) para as variáveis numéricas. A análise de normalidade foi feita pelo teste de Shapiro-Wilk. As variáveis numéricas foram dicotomizadas pela mediana. A associação entre o uso de MPII e as variáveis independentes foi calculada utilizando o teste do χ^2^ e o teste exato de Fisher, sendo considerados estatisticamente significante os resultados de p<0,05. As variáveis independentes, que apresentaram associação com uso de MPII e obtiveram valor de p<0,20 na análise univariada, foram listadas para a regressão logística multivariada. Para obtenção do modelo final, utilizou-se o método *backward stepwise* , permanecendo as variáveis com valor p<0,05. Na avaliação da adequação do modelo final, empregou-se o teste de Hosmer e Lemeshow (adequado se p>0,05).

## RESULTADOS

As características clínicas e sociodemográficas dos 255 idosos participantes do estudo estão descritas na tabela 1 . A amostra foi constituída principalmente por mulheres (57,3%), a mediana de idade foi de 75 anos (intervalo interquartil − IQI =13) e a maioria dos participantes não possuía companheiro (50,9%).

**Tabela 1:** Características sociodemográficas e clínicas

Características sociodemográficas	
Idade, anos	75 [13]
Sexo feminino	146 (57,3)
Não tem companheiro	130 (50,9)
Características clínicas	
Escore VES-13^*^	5 (6,0)
Índice de comorbidade de Charlson	5 (2,0)
Diagnóstico de admissão	
Doenças do aparelho respiratório	64 (25,1)
Doenças do aparelho geniturinário	43 (16,9)
Doenças do aparelho circulatório	30 (11,8)
Sintomas, sinais e achados anormais de exames clínicos e de laboratório, não classificados em outra parte	25 (9,8)
Algumas doenças infecciosas e parasitárias	21 (8,2)
Doenças do aparelho digestivo	18 (7,1)
Transtornos mentais e comportamentais	14 (5,5)
Doenças endócrinas, nutricionais e metabólicas	11 (4,3)
Outras	40 (15,6)
Farmacoterapia	
Polifarmácia (≥5 medicamentos)	174 (68,2)
Número de medicamentos em uso na alta hospitalar	6 [4]
Número de medicamentos em uso na admissão	5 [5]
Pacientes em uso de MPII	149 (58,4)
Número máximo de MPII por paciente	4 (1-4)

Resultados expressos por mediana [amplitude interquartil], n (%) ou n (mínimo-máximo). * Indivíduos cujas pontuações são ≥3 apresentam risco 4,2 vezes maior de declínio funcional e morte em 2 anos, quando comparados àqueles com escores menores. VES-13: *Vulnerable Elders Survey* ; MPII: medicamentos potencialmente inapropriados para idosos.

A mediana do escore de vulnerabilidade foi igual 5 pontos (IQI=6). A carga de morbidade avaliada pelo ICC apresentou mediana igual a 5 (IQI=2). As doenças do aparelho respiratório (25,1%), seguidas das do aparelho geniturinário (16,9%) e circulatório (11,8%), foram os principais diagnósticos clínicos na admissão.

No que se refere às características farmacoterápicas, a mediana do número de medicamentos em uso na admissão foi 5 (IQI=5), sendo esse valor superior na alta hospitalar (mediana=6; IQI=5). Houve predominância de idosos em uso de polifarmácia (68,2%).

O uso de MPII na alta hospitalar foi verificado em 58,4% dos idosos entrevistados. O número mínimo e máximo de MPII prescritos na alta variou de 1 a 4, respectivamente.

Os inibidores de bomba de prótons (IBP) foram a classe de MPII mais utilizada (43,8%). Outros MPII frequentes foram os benzodiazepínicos (14,9%), antipsicóticos de segunda geração (14,9%), fenobarbital (3,8%) e haloperidol (3,4%). A distribuição das frequências de utilização de MPII está demonstrada na tabela 2 .

**Tabela 2:** Medicamentos potencialmente inapropriados para idosos (MPII), independente da condição clínica e de acordo com Consenso Brasileiro de Medicamentos Potencialmente Inapropriados para Idosos

Critério/MPII	n (%)
Inibidores de bomba de prótons (>8 semanas)	91 (43,8)
Omeprazol	77 (37,0)
Pantoprazol	14 (6,7)
Benzodiazepínicos	31 (14,9)
Clonazepam	21 (10,1)
Lorazepam	8 (3,8)
Bromazepam	8 (3,8)
Alprazolam	1 (0,5)
Antipsicóticos de segunda geração	31 (14,9)
Quetiapina	17 (8,2)
Risperidona	12 (5,8)
Clozapina	2 (1,0)
Barbitúricos	
Fenobarbital	8 (3,8)
Antipsicóticos de primeira geração	
Haloperidol	7 (3,4)
Antiarrítmicos classes Ia, Ic, III	
Amiodarona	6 (2,9)
Hipnóticos não benzodiazepínicos por tempo >90 dias	
Zolpidem	4 (1,9)
Antidepressivos tricíclicos terciários	4 (1,9)
Nortriptilina	3 (1,4)
Amitriptilina	1 (0,5)
Nitrofurantoína	4 (1,9)
Uso prolongado de opioides fortes como terapia de primeira linha para dor leve/moderada	4 (1,9)
Morfina	3 (1,4)
Metadona	1 (0,5)
Glibenclamida	3 (1,4)
Alfa agonistas de ação central	
Clonidina	3 (1,4)
Metoclopramida	3 (1,4)
Uso prolongado de colchicina para tratamento de gota	2 (1,0)
Anti-inflamatórios não esteroidais (não seletivos COX)	2 (1,0)
Ibuprofeno	1 (0,5)
Naproxeno	1 (0,5)
Anti-histamínicos de primeira geração	
Dexclorfeniramina	2 (1,0)
Antiparkinsonianos com forte ação anticolinérgica	
Biperideno	1,0 (0,5)
Óleo mineral (via oral)	1,0 (0,5)
Relaxantes musculares	
Ciclobenzaprina	1,0 (0,5)

COX: ciclo-oxigenase.

O uso de MPII esteve associado ao sexo (feminino), à internação de origem (geriatria), à presença de doenças (hipertensão arterial sistêmica e depressão), à polifarmácia e ao índice de complexidade da farmacoterapia alto, conforme demonstrado pela análise estatística univariada ( Tabela 3 ).

**Tabela 3:** Análise univariada e multivariada dos fatores associados à utilização de medicamentos potencialmente inapropriados entre idosos atendidos em um hospital público de Minas Gerais

Variável	Uso de MPII					
	Frequência*		Análise univariada		Análise multivariada*	
		
	Sim	Não	Razão de chances	Valor de p	Razão de chances	Valor de p
	n (%)	n (%)	(IC95%)		(IC95%)	
Sociodemográficas						
Sexo						
Feminino	94 (63,1)	52 (49,1)	1,775 (1,070-2,943)	0,026	-	-
Masculino	55 (36,9)	54 (50,9)	1			
Idade						
≥75	84 (56,4)	56 (52,8)	1,154 (0,700-1,903)	0,575	-	-
<75	65 (43,6)	50 (47,2)	1			
Clínicas						
Internação de origem						
Geriatria	66 (44,3)	31 (29,2)	0,520 (0,306-0,882)	0,015	0,513 (0,295-0,892)	0,018
Outras clínicas	83 (55,7)	75 (70,8)	1			
AVE						
Sim	22 (14,8)	15 (14,2)	1,051 (0,517-2,136)	0,891	-	-
Não	127 (85,2)	91 (85,8)	1			
Insuficiência cardíaca						
Sim	24 (16,1)	14 (13,2)	1,262 (0,619-2,571)	0,522	-	-
Não	125 (83,9)	92 (86,8)	1			
DPOC						
Sim	20 (13,4)	14 (13,2)	1,019 (0,489-2,122)	0,960	-	-
Não	129 (86,6)	92 (86,8)	1			
Câncer						
Sim	19 (12,8)	10 (9,4)	1,403 (0,624-3,154)	0,411	-	-
Não	130 (87,2)	96 (90,6)	1			
*Diabetes mellitus*						
Sim	63 (42,3)	47 (44,3)	0,920 (0,556-1,520)	0,744	-	-
Não	86 (57,7)	59 (55,7)	1			
Pneumonia						
Sim	32 (21,5)	21 (19,8)	1,107 (0,597-2,052)	0,747	-	-
Não	117 (78,5)	85 (80,2)	1			
Demência						
Sim	51 (34,2)	28 (26,4)	1,450 (0,838-2,509)	0,184	-	-
Não	98 (65,8)	78 (73,6)	1			
Doença renal crônica						
Sim	31 (20,8)	14 (13,2)	1,726 (0,868-3,433)	0,117	-	-
Não	118 (79,2)	92 (86,8)	1			
Fibrilação atrial						
Sim	12 (8,1)	9 (8,5)	0,944 (0,383-2,328)	0,900	-	-
Não	137 (91,9)	97 (91,5)	1			
HAS						
Sim	113 (75,8)	68 (64,2)	1,754 (1,016-3,029)	0,043	-	-
Não	36 (24,2)	38 (35,8)	1			
Infarto agudo do miocárdio						
Sim	14 (9,4)	5 (4,7)	2,095 (0,731-6,005)	0,16^†^	-	-
Não	135 (90,6)	101 (95,3)	1			
Parkinsonismo						
Sim	7 (4,7)	0 (0,0)	1,746 (1,568-1,945)	0,44^†^	-	-
Não	142 (95,3)	106 (100)				
Depressão						
Sim	35 (23,5)	11 (10,4)	2,652 (1,278-5,503)	0,007	2,208 (1,035-4,707)	0,040
Não	114 (76,5)	95 (89,6)	1			
Índice de comorbidade de Charlson						
≥5	85 (57,0)	58 (54,7)	1,099 (0,666-1,815)	0,712	-	-
<5	64 (43,0)	48 (45,3)				
Funcionalidade						
VES-13						
≥5	85 (57,0)	51 (48,1)	1,432 (0,868-2,362)	0,159	-	-
<5	64 (43,0)	55 (51,9)	1			
Farmacoterápicas						
Polifarmácia 5						
Sim	114 (76,5)	60 (56,6)	2,497 (1,456-4,283)	0,001	2,495 (1,431-4,349)	0,001
Não	35 (23,5)	46 (43,4)	1			
Índice de complexidade da farmacoterapia alto						
Sim	93 (62,4)	40 (37,7)	2,740 (1,639-4,581)	0,000	-	-
Não	56 (37,6)	66 (62,3)	1			

^†^Valor de p foi calculado pelo teste exato de Fisher; * teste da qualidade de ajuste de Hosmer-Lemeshow: graus de liberdade = 5; χ^2^= 2,40; valor de p=0,79. MPII: medicamentos potencialmente inapropriados para idosos; AVE: acidente vascular encefálico; DPOC: doença pulmonar obstrutiva crônica; HAS: hipertensão arterial sistêmica; VES-13: *Vulnerable Elders Survey* .

No modelo de regressão logística final da análise multivariada, a internação de origem (geriatria), a presença de depressão e a polifarmácia apresentaram associação positiva com uso de MPII. Ficar internado na clínica geriátrica mostrou associação inversa com o uso de MPII.

## DISCUSSÃO

A prevalência de MPII na alta hospitalar entre os idosos investigados foi elevada e esteve associada à presença de depressão e uso de polifarmácia. A internação em unidade de geriatria apresentou associação inversa com uso de MPII. O estudo contribuiu para ampliar o conhecimento da dimensão da utilização de MPII na alta hospitalar, pois investigação prévia realizada no Brasil foi limitada a pacientes com histórico de doença cardiovascular. Outra contribuição do estudo foi a identificação dos fatores associados. A frequência de utilização de MPII foi superior à de investigação realizada em hospital de ensino de São Paulo^19^ (13,9%) e às de países europeus, que variaram de 23,5 a 48%.^12 , 20 , 21^ Um estudo brasileiro também demonstrou alta prevalência de uso de MPII durante a hospitalização,^22^ evidenciando a importância de, desde a internação, buscar medidas para prevenir e reduzir o uso desses medicamentos. As comparações dos resultados devem ser realizadas com cautela devido às diferenças metodológicas e ao critério explícito empregado na identificação de MPII.

O presente estudo é o primeiro a determinar a frequência de uso de MPII na alta hospitalar empregando o CBMPII. Estudos utilizando critérios explícitos desenvolvidos na perspectiva da realidade de um país são importantes, pois propiciam melhor descrição do perfil de utilização de MPII e a identificação dos fatores associados. Além disso, proporcionam bases para a prática clínica segura e para adoção de políticas públicas que visam reduzir agravos relacionados à farmacoterapia nos idosos do país. O CBMPII apresentou boa concordância com os critérios internacionais AGS Beers 2015 e *European Union List of Potentially Inappropriate Medications* (EU)(7)-PIM *List* ), evidenciando sua factibilidade para uso em investigações farmacoepidemiológicas e na prática clínica.^23^

Um achado relevante foi a alta prevalência de IBP na prescrição de alta hospitalar dos idosos. Esse resultado está de acordo com pesquisa realizada em hospital comunitário japonês, utilizando o AGS Beers 2015, cuja frequência de IBP correspondeu a cerca de 38% dos MPII utilizados na alta hospitalar.^11^ O uso prolongado (>8 semanas) de IBP eleva o risco de infecções por *Clostridium difficile* ,^6^ aumenta a propensão de fraturas, reduz a densidade mineral óssea,^6 , 7^ aumenta o risco de demência e de insuficiência renal.^7^ Vale destacar que estudo brasileiro com idosos da comunidade também demonstrou alta prevalência de uso de IBP.^23^ Tais achados reforçam a importância da elaboração de guias para orientar a prescrição adequada e segura de IBP, assim como a definição de diretrizes para subsidiar a desprescrição dessa classe terapêutica.

Ressalta-se, também, a frequência elevada de prescrições contendo benzodiazepínicos. A alta prevalência de uso dos benzodiazepínicos entre a população geriátrica já é bem estabelecida. No Japão, cerca de 30% dos pacientes receberam prescrição de benzodiazepínicos na alta hospitalar.^11^ Nos Estados Unidos, estudo que avaliou prescrições ambulatoriais por um período de 10 anos revelou que os benzodiazepínicos foram prescritos para idosos em 12,45% das consultas.^24^ O uso de benzodiazepínicos por idosos está associado a desfechos negativos, como quedas, fraturas, acidentes automobilísticos, comprometimento cognitivo e *delirium,* ^6 , 7^ sobretudo os de ação prolongada.^6^

Entre os medicamentos inapropriados com atuação no sistema nervoso central, na casuística investigada, identificou-se alta prevalência de uso de antipsicóticos – típicos (haloperidol) e atípicos (quetiapina, risperidona e clozapina) –, padrão semelhante ao encontrado em estudos realizados no exterior.^11 - 21^ Essa classe terapêutica é frequentemente prescrita para tratamento de sintomas comportamentais e psicológicos da demência no idoso. Os antipsicóticos são considerados MPII por aumentarem o risco de acidente vascular cerebral,^6 , 7^ de declínio cognitivo^6^ e mortalidade em idosos em uso prolongado.^6^

Nos idosos, o uso de barbitúricos deve ser evitado, pelo elevado potencial de dependência física e tolerância na indução do sono, além do risco de intoxicação, devido ao índice terapêutico estreito.^6 , 7^ O aumento na permeabilidade da barreira hematoencefálica, associado a outras modificações fisiológicas do processo de envelhecimento, eleva a sensibilidade aos barbitúricos^25^ e justifica a parcimônia em sua prescrição. O uso de barbitúricos entre os idosos, incluídos no estudo, foi restrito a fenobarbital e com frequência reduzida.

Assim, estratégias para evitar a prescrição e o uso de MPII devem ser implementadas. A assistência prestada por farmacêuticos clínicos em associação a uma equipe multidisciplinar de cuidados geriátricos tem sido descrita como a prática clínica efetiva para melhorar a adequação da prescrição e garantir a segurança da farmacoterapia de idosos na admissão e na alta hospitalar.^26^

A polifarmácia demonstrou associação positiva com uso de MPII na alta hospitalar, resultado semelhante ao encontrado em outros estudos.^5 - 13^ Uma investigação espanhola revelou que, para cada medicamento adicional na alta hospitalar, aumenta-se o risco de uso de MPII em 14 ou 15%.^5^ Apesar dos desfechos negativos, a polifarmácia, em muitos casos, mostra-se necessária. O tratamento de múltiplas doenças exige o uso concomitante de diversos medicamentos, sendo a polifarmácia estratégia para a manutenção adequada da farmacoterapia do idoso.^27^

Associação positiva foi também encontrada para possuir depressão e uso de MPII. Tal achado pode ser atribuído à elevada prevalência de psicofármacos entre os MPII utilizados pela população investigada. Uma coorte francesa que avaliou idosos hospitalizados encontrou associação entre o uso prévio de psicofármacos (2 semanas antes da hospitalização) e a presença de depressão.^28^ No Brasil, a depressão foi a variável mais fortemente associada ao uso de psicofármacos por idosos residentes na região metropolitana de Belo Horizonte (MG).^29^

A internação na unidade de clínica geriátrica mostrou-se fator protetor para o uso de MPII na casuística investigada. O cuidado prestado em unidade geriátrica especializada está associado a benefícios clínicos, como redução da polifarmácia,^30^ simplificação dos esquemas posológicos^30^ e menor utilização de MPII na alta hospitalar.^10^ Esse resultado reforça a importância de uma abordagem global e multidisciplinar do cuidado do idoso, visando ao reconhecimento das particularidades e das demandas do envelhecimento − em especial, aos fatores associados ao uso de medicamentos.^26^

O presente estudo apresenta resultados semelhantes aos encontrados na literatura internacional e representa um marco nos estudos farmacoepidemiológicos envolvendo idosos no Brasil, pois utiliza um critério nacional. Entretanto, apresenta limitações que merecem ser destacadas. A primeira é a ausência de identificação do tempo de uso e da dose dos medicamentos, o que pode interferir na mensuração de determinados critérios de MPII. Além disso, a avaliação do uso de MPII sem considerar a condição clínica pode ter contribuído para superestimar a frequência de utilização de MPII. A segunda limitação é não propiciar generalizações em função da realização em um único hospital, cuja clientela é restrita a servidores públicos.

## CONCLUSÃO

A frequência de utilização de medicamentos potencialmente inapropriados para idosos na alta do hospital investigado foi alta. Polifarmácia e depressão foram fatores positivamente associados ao uso destes medicamentos. Ficar internado em unidade geriátrica apresentou associação inversa com o uso de medicamentos potencialmente inapropriados para idosos. Estratégias para melhorar a farmacoterapia do idoso devem ser implementadas visando à qualidade assistencial e à segurança na transição do cuidado.
